# Addressing ethnic and global health inequalities in the era of artificial intelligence healthcare models: a call for responsible implementation

**DOI:** 10.1177/01410768231187734

**Published:** 2023-07-19

**Authors:** Mohammad R Ali, Claire A Lawson, Angela M Wood, Kamlesh Khunti

**Affiliations:** 1 Department of Cardiovascular Sciences, University of Leicester, Leicester, LE1 7RH, UK; 2 Cardiovascular Epidemiology Unit, Department of Public Health and Primary Care, Victor Phillip Dahdaleh Heart and Lung Research Institute, 2152 University of Cambridge, Cambridge, CB2 0SR, UK; 3 Ethnic Health Research, Diabetes Research Centre, 4488 University of Leicester, Leicester, LE5 4PW, UK

Artificial intelligence (AI) tools are being used more frequently in healthcare, particularly in medical applications such as radiology and risk prediction.^1^ With this comes the palpable excitement to democratise healthcare using new AI models such as ChatGPT. However, we must tread carefully and act urgently to ensure that these large language models (LLMs) and their iterations do not exacerbate recently highlighted ethnic and global health inequalities.

The biases in AI systems often stem from their training data. For LLMs, this training process starts with ‘pre-training’: a large corpus of data scraped and ingested by the model from disparate sources such as websites and scientific literature. Using these data, a technique called ‘self-supervised learning’ predicts words based on the preceding word it has written. These models can then be fine-tuned for specific tasks/domains.^2^ However, due to potential risks it poses to societal safety, concerns have been raised about opacity of the data sources used to train LLMs.^3^ A particularly dangerous prospect for healthcare LLMs is their susceptibility to ‘hallucinations’ – creating ungrounded, subtly incorrect information without self-awareness.^4,5^

Given the nature of these training processes, there is a risk that health inequalities may become even more entrenched, particularly for ethnic minorities, due to systemic structural and societal biases in healthcare research perpetuated when training LLMs. Evidence depicts that ethnicity data are often missing^6^ or unrecorded due to privacy concerns.^7^ Compounding this is the inability to define an accurate scale of how little ethnic minorities participate in research.^8^ The broader generalisability of research findings is already concerning as participants are less representative than those receiving the evaluated interventions in trials.^9^ The scarcity of published research and therefore smaller sample size with ethnic minorities will mean ‘less certainty’ statistically and, thus, less accuracy regarding effect sizes of interventions. Furthermore, this disproportionately lower representation of ethnic minorities in research has evidence of causing harm, e.g. by creating ineffective drug treatments^10^ or treatment guidance which could be regarded as racist.^11^ It is widely accepted that a differential risk is associated with being from an ethnic minority background across many disease groups.^12^ If the published literature already contains biases and less precision, it is logical that future AI models will maintain and further exacerbate them.

Beyond the issues of ethnic inequalities, there is a significant concern that health inequalities could worsen in low- and middle-income countries (LMICs), which suffer from most chronic disease without the requisite resources. AI models are primarily developed in wealthier nations like the USA and Europe, collectively known as the Global North, and a significant disparity in research and development exists between high- and low-income countries.^13^ Most published research does not prioritise the needs of those in the LMICs with their unique health challenges, particularly around healthcare provision. LLMs, using published literature, may provide advice based on the corpus of data trained on populations wholly different from those in LMICs. Inevitably, the outputs of these and any future AI models being widely generalisable and truly inclusive will be limited.

While crucial to acknowledge these potential difficulties, it is equally important to focus on solutions. We must exercise caution acknowledging we cannot and should not stem the flow of progress. Nevertheless, we ask researchers to consider the broader generalisability of AI models including LLMs. Several actions need to be implemented to overcome potentially exacerbating health inequalities.

First, AI models should clearly describe their body of medical data used in model development's ‘pre-training’ and refinement stages, including ethical approvals. Second, work is needed to address ethnic health inequalities in research. This includes recommendations to improve recruitment and recording of ethnicity information.^8,9^ Encouraging wider participation and recording of ethnicity data will enable AI models to be trained on representative populations. Third, we must ensure that the data used to train the AI model are adequately representative while considering potential model biases and its accuracy. The model must consider key factors, including ethnicity, age, sex and socioeconomic factors. Fourth, using appropriate methodologies, further research is required to understand the generalisability of LLMs and other AI models in ethnically diverse populations. Researchers should consider recalibrating models before using them in ethnically diverse populations. Moreover, when utilising published research, it is advisable to assess the risk of bias^14^ and research quality using tools like GRADE.^15^ Given their reliance solely on the training data coupled with their inability to reason, these models have limited capability in discerning between ‘good’ and ‘poor’ research. By ensuring that only high-quality research is used for training AI models, we can reduce the risk of providing incorrect answers.

In summary, LLMs and future AI models have the potential for transforming healthcare. However, caution is warranted before these models are used in healthcare with ethnic minority populations. The corpus of data in training any future AI models must represent the population they are deployed, to mitigate current research trends that show it is unrepresentative of different populations. By addressing these considerations, we can harness the power of AI models to drive positive change in healthcare while promoting fairness and inclusivity.

## Declarations

### Guarantor

MRA is the guarantor. He accepts full responsibility for the work.

### Provenance

Not commissioned; editorial review.
